# Joint Beamforming Design and User Clustering Algorithm in NOMA-Assisted ISAC Systems

**DOI:** 10.3390/s24206633

**Published:** 2024-10-15

**Authors:** Qingqing Yang, Runpeng Tang, Yi Peng

**Affiliations:** 1 School of Information Engineering and Automation, Kunming University of Science and Technology, Kunming 650500, China; 20090119@kust.edu.cn (Q.Y.); 15182477730@163.com (R.T.); 2 Yunnan Provincial Key Laboratory of Computer Science, Kunming University of Science and Technology, Kunming 650500, China

**Keywords:** integrated sensing and communications, non-orthogonal multiple access, user clustering, beamforming design, fractional programming

## Abstract

To enhance the performance of non-orthogonal multiple access (NOMA)-assisted integrated sensing and communication (ISAC) systems in multi-user distributed scenarios, an improved Gaussian Mixture Model (GMM)-based user clustering algorithm is proposed. This algorithm is tailored for ISAC systems, significantly improving bandwidth reuse gains and reducing serial interference. First, using the Sum of Squared Errors (SSE), the algorithm reduces sensitivity to the initial cluster center locations, improving clustering accuracy. Then, direction weight factors are introduced based on the base station position and a penalty function involving users’ Euclidean distances and sensing power. Modifications to the EM algorithm in calculating posterior probabilities and updating the covariance matrix help align user clusters with the characteristics of NOMAISAC systems. This improves users’ interference resistance, lowers decoding difficulty, and optimizes the system’s sensing capabilities. Finally, a fractional programming (FP) approach addresses the non-convex joint beamforming design problem, enhancing power and channel gains and achieving co-optimizing sensing and communication signals. The simulation results show that, under the improved GMM user clustering algorithm and FP optimization, the NOMA-ISAC system improves user spectral efficiency by 4.3% and base station beam intensity by 5.4% compared to traditional ISAC systems.

## 1. Introduction

With the rapid development of technologies such as the Internet of Things (IoT), augmented reality (AR), and virtual reality (VR), traditional communication methods can no longer meet the growing demands for interaction. Users now expect more immersive and intuitive ways to access and communicate information beyond simple texts and images. This shift imposes higher requirements on next-generation wireless communication systems like B5G (Beyond Fifth Generation) and 6G (Sixth Generation) [1]. In addition to offering higher data transmission rates and more reliable mobile connections, next-generation wireless systems will enable emerging applications such as smart cities, VR, smart homes, and intelligent transportation. Integrated sensing and communications (ISAC) is widely regarded as one of the practical solutions to achieve this goal [2]. ISAC integrates wireless communication and radar sensing, sharing the same spectrum resources and hardware platforms [3], thus alleviating the issue of spectrum scarcity. Therefore, exploring and applying ISAC technology holds significant social, economic, and technical value.

Non-orthogonal multiple access (NOMA), as one of the critical technologies in 5G, allows users to share time–frequency resources through power domain multiplexing [4]. The transmitter superimposes the signals of different users on the same frequency band and distinguishes them by allocating power and coding. This non-orthogonal signal superposition effectively improves spectrum efficiency, meeting multi-user access requirements while reducing system complexity [5]. The receiver uses successive interference cancellation (SIC) to process multiple users’ signals sequentially, eliminating interference and decoding them accordingly. Many studies are currently focusing on applying NOMA in integrated sensing and communication (ISAC). In [6], the NOAM-ISAC framework was first established, and NOMA-assisted joint radar and multicast–unicast communication (Rad-MU-Com) was proposed. A hybrid multicast–unicast message transmission between radar and communication users was enabled using a multiple-input multiple-output (MIMO) dual-function radar-communication (DFRC) base station. In [7], the NOMA-based ISAC framework optimized beamforming design, enhancing communication throughput and sensing power. In [8], the NOMA-based joint communication, sensing, and multi-layer computing (JCSMC) framework optimized resource allocation for efficient computation offloading.

These studies emphasize the improvement of the integrated sensing and communication (ISAC) system performance through NOMA; however, the system performance under NOMA communication largely depends on the interference intensity between users and the difficulty of SIC decoding, which relies on user clustering [9]. The authors of [10] reduce user interference through an efficient user clustering algorithm considering different transmission power scenarios and provide a robust power allocation (PA) solution under imperfect CSI assumptions. The authors of [11] propose a low-complexity learning-based user clustering method using an improved mean-shift clustering algorithm, which effectively utilizes the degrees of freedom in the system to form NOMA clusters and significantly enhances performance. The authors of [12] investigate user clustering and power control in MISO-NOMA networks. The study proposes a two-step user clustering and power control algorithm, with the proposed K-means-based iterative power control scheme significantly outperforming other reference methods regarding power consumption and energy efficiency. The authors of [13] address the issues of high node death rates and energy consumption in energy-efficient clustered routing communication for wireless sensor networks, proposing an energy-efficient clustered routing algorithm based on an energy iterative model and swarm optimization. The authors of [14] employ Gaussian Mixture Models (GMMs) for the unsupervised clustering of received signals, optimizing decision boundaries to improve bit error rate (BER) performance.

However, the clustering algorithms in the literature above are rarely applied in NOMA-ISAC systems. The K-means algorithm requires each cluster to have a regular shape, and its high sensitivity to initial values, like that of the GMM algorithm, makes it challenging to significantly improve the performance of NOMA-ISAC systems. Moreover, most existing user clustering algorithms [15] group users with close Euclidean distances, which contradicts the NOMA clustering approach that aims to maximize differences in user channel gains. The authors of [16] introduce a dynamic user clustering method based on CDA and COA algorithms, using a joint optimization approach to maximize total rate and sensing capability. Although this algorithm has some applications in ISAC, it is not closely related to sensing, and very few user clustering algorithms are applied in NOMA-ISAC systems. To date, no scholars have employed clustering algorithms to optimize the performance of integrated sensing and communication systems in multi-user distribution scenarios. This paper proposes a reliable user clustering algorithm suitable for NOMA-ISAC systems.

To further enhance the performance of the integrated sensing and communication (ISAC) system and address the non-convex problem of beamforming design, this paper utilizes a dual-function base station that can simultaneously achieve communication and sensing functions. The improved Gaussian Mixture Model (GMM) clustering algorithm forms strip-shaped clusters that adapt to NOMA-ISAC, simultaneously simplifying the objective function. Additionally, due to the difficulty in obtaining stable solutions for the multi-rate problem involved in the objective function and the high coupling of multiple objective variables, to address the integrated sensing and communication beamforming design problem, fractional programming is used to transform the maximization of the logarithmic and fractional composite spectral efficiency problem into a series of convex problems. After obtaining the simplified objective function using the cluster splitting algorithm, a suboptimal solution to the original objective function can be obtained by fixing the auxiliary variables [17] and taking the partial derivatives of the reconstructed problem.

The main contributions of this paper can be summarized as follows:

An improved GMM user clustering algorithm is proposed for multi-user NOMA-assisted ISAC systems as follows:

1. Sensitivity to initial centroid positions is reduced by computing the Sum of Squared Errors (SSE).
2. Directional weight factors and penalty functions are introduced to ensure the algorithm forms strip-like clusters suited to the NOMA-ISAC system.
3. The posterior probability in the GMM algorithm is modified, and an adaptive covariance matrix update mechanism is used to optimize the coordination between communication and sensing.
4. The improved GMM user clustering algorithm reduces inter-user interference in traditional multiple-access methods and increases bandwidth reuse gain.
5. Fractional programming handles the joint beamforming design for ISAC systems. Converting the maximization of spectral efficiency involving logarithmic and fractional terms into a series of convex problems achieves more effective system power gain and channel gain.

## 2. System Model

**Figure 1:**
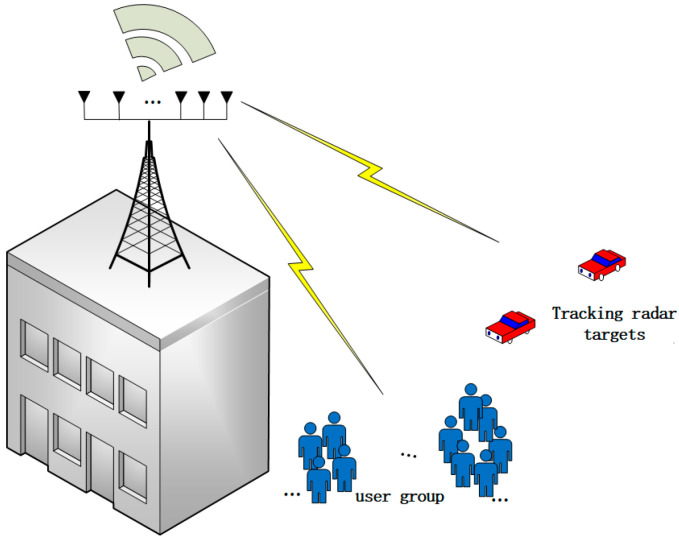
Integrated sensing and communication system model.

The integrated sensing and communication system model assisted by NOMA is illustrated in Figure 1, featuring a dual-functional base station that simultaneously performs communication and sensing. The base station is equipped with N antennas, utilizing a Uniform Linear Array (ULA) to serve K single-antenna users and Q sensing targets, ensuring minimal interference between antenna units and maintaining good phase alignment. The spacing between each antenna is set to half the wavelength. Users are unevenly distributed around the base station, while the sensing targets are considered line-of-sight links relative to the base station. All users are eventually divided into M clusters, with the m−th cluster denoted as $C_{m},m\in \{1,2,\ldots ,M\}$. $\left|C_{m}\right|$ is the number of users within the m−th cluster and satisfies $\left|C_{1}\right|+\left|C_{2}\right|,\ldots ,+\left|C_{M}\right|=K$. After clustering, users within each cluster utilize NOMA to share time–frequency resources. Users are distinguished through power multiplexing. Inter-cluster communication employs orthogonal multiple access (OMA). Each cluster is orthogonal. Interference between clusters is ignored to simplify system analysis and design.

### 2.1. Communications Model

During downlink communication with NOMA, the signal received by the i−th user $u_{i}^{m}$ of the m−th cluster can be expressed as the following:

(1) $$y_{i}^{m}=h_{i}^{m}\sum_{i\in k}w_{i}^{m}s_{i}^{m}+\sum_{\tilde{i}\in \left|C_{m}\right|/\{i\}}h_{i}^{m}w_{\tilde{i}}^{m}s_{\tilde{i}}^{m}+n_{i}$$

where k={1,2,…,K}, $w_{i}s_{i}$ represents the superimposed signal transmitted by the base station, $w_{i}$ denotes the beamforming vector of the user $u_{i}^{m}$, $s_{i}$ denotes the information data stream of the user $u_{i}^{m}$, $h_{i}^{m}$ denotes the channel gain from the base station to the user $u_{i}^{m}$, $\tilde{i}\in \left|C_{m}\right|/\{i\}$ denotes the remaining users other than the user $u_{i}^{m}$ in the m−th cluster and $n_{i}$ denotes additive Gaussian white noise with a variance of $\sigma^{2}$.

In the system model of this paper, the link from the base station to the user is considered a line-of-sight (LOS) link. Therefore, the free-space path loss model is used as the path loss model for the user, which is given explicitly by the following:

(2) $$\Gamma_{i}^{m}=32.5+36.7\mathrm{lg}d_{i}^{m}$$

where $d_{i}^{m}$ represents the distance from the user $u_{i}^{m}$ to the base station. Let the position of the base station be denoted by $b=\left(b_{x},b_{y}\right)$ and the position of the user $u_{i}^{m}$ be denoted by $u_{i}^{m}=\left(i_{x},i_{y}\right)$, where $\left(b_{x},b_{y}\right)$ and $\left(i_{x},i_{y}\right)$ denote the horizontal and vertical coordinates of the base station and user $u_{i}^{m}$, respectively. Then, $d_{i}^{m}$ can be expressed as the following:

(3) $$d_{i}^{m}=\sqrt{{\left(b_{x}-i_{x}^{m}\right)}^{2}+{\left(b_{y}-i_{y}^{m}\right)}^{2}}$$

Without loss of generality, let the distances from the users in the m−th cluster to the base station be sorted as $d_{\left|C_{m}\right|}^{m}>,\ldots ,>d_{1}^{m}$. The channel gain for users within the cluster can thus be expressed as $h_{1}^{m}>,\ldots ,>h_{\left|C_{m}\right|}^{m}$. Based on the above channel gain condition, the signal-to-interference-plus-noise ratio (SINR) for user $u_{i}^{m}$ in cluster m is given as follows:

(4) $$\gamma_{i}^{m}=\frac{{\left|w_{i}^{m}h_{i}^{m}\right|}^{2}}{\sum_{j=1}^{i-1}{\left|w_{j}^{m}h_{i}^{m}\right|}^{2}+\sigma^{2}}$$

where $\sum_{j=1}^{i-1}{\left|w_{j}^{m}h_{i}^{m}\right|}^{2}$ is the interfering signal that the SIC decodes and fails to eliminate. The reachable rate of user $u_{i}^{m}$ can be expressed as the following:

(5) $$r_{i}^{m}=\frac{B}{M}\log_{2}\left(1+\frac{{\left|w_{i}^{m}h_{i}^{m}\right|}^{2}}{\sum_{j=1}^{i-1}{\left|w_{j}^{m}h_{i}^{m}\right|}^{2}+\frac{B}{M}\sigma^{2}}\right)$$

where B is the system bandwidth. The sum rate of all users is expressed as the following:

(6) $$R_{sum}=\sum_{m=1}^{M}\sum_{i=1}^{\left|C_{m}\right|}r_{i}^{m}$$

In NOMA communication, complex user channel conditions are one of the critical factors affecting the sum rate. By increasing the channel gain difference between users sharing the same channel, the difficulty of SIC decoding and user interference can be reduced. This, in turn, improves the spectral efficiency for users.

### 2.2. Sensing Model

In an ISAC system, the communication waveform can be used for user communication and sensing detection. To achieve good sensing performance, the goal of the sensing system is to maximize the sensing power under the condition of prior target information [16]. The sensing power is expressed as follows:

(7) $$p(\theta_{q})=a^{H}(\theta_{q})R_{w}a(\theta_{q})$$

where $\theta_{q}$ is the direction of the sensing target, $a(\theta_{q})=\frac{1}{\sqrt{N}}{\left[1,e^{j\frac{2\pi }{\lambda }d\sin(\theta_{q})},\ldots ,e^{j\frac{2\pi }{\lambda }(N-1)d\sin(\theta_{q})}\right]}^{T}$ is the steering vector in the direction of the target, and $R_{w}=w_{i}w_{i}^{H}$ is the covariance matrix of the transmitted signal. In the waveform design for sensing, this corresponds to designing the covariance matrix of the transmitted signal [18].

Constant modulus constraints are typically introduced to improve transmitter efficiency and enable the radar’s nonlinear amplifiers to operate at maximum efficiency, enhancing radar transmission performance. However, in practical operation, there may be a conflict between radar detection and estimation performance. Therefore, the Peak Average Power Ratio (PAPR) is typically introduced to address more general energy constraint issues. The mathematical definition of PAPR is as follows:

(8) $$PAPR(W)=\frac{\underset{n}{\max{\left|W(n)\right|}^{2}}}{\frac{{\left\|W\right\|}^{2}}{N}}\leq \eta$$

where $W=vec(w)\in {\mathbb{C}}^{N\times 1},n=1,2,\ldots ,N$. When η=0, this corresponds to the constant envelope constraint. When η=1, the peak-to-average ratio is converted into the continuous modulus constraint. The authors of [19] have verified that relaxing the constant envelope constraint can yield better performance for the transmitted waveform. Moreover, the peak-to-average constraint is more flexible than the constant modulus constraint, providing more degrees of freedom for the sensing waveform.

## 3. Problem Description

In the ISAC system model, considering the trade-off between communication and sensing performance, a trade-off factor ρ is introduced to balance the system’s performance of communication and sensing. Under this condition, the joint waveform design problem for the ISAC system can be described as follows:

(9) $$\begin{array}{l} F1:\max_{w_{i},\upsilon_{i}^{m},r_{i}^{m}}f\left(r_{i}^{m},w_{i}\right)=\rho \sum_{m=1}^{M}\sum_{i=1}^{\left|C_{m}\right|}\upsilon_{i}^{m}r_{i}^{m}+(1-\rho )\sum_{q\in \mathbb{Q}}p(\theta_{q})\\ s.t.\;C1:\;r_{i}\geq r_{\min,i},\forall i\in k\\ \;C2:\;\sum_{m=1}^{M}\upsilon_{i}^{m}=1,\upsilon_{i}^{m}\in \{0,1\},i\in \{1,2\ldots ,\left|C_{m}\right|\}\\ \;C3:\;W^{H}W=P_{t}\\ \;C4:\;W^{H}E_{n}W\leq \frac{P_{t}\eta }{N},n=1,2,\ldots ,N \end{array}$$

where ρ∈[0,1] is the trade-off factor for balancing communication and sensing performances. When ρ=0, the system considers only sensing performance. When ρ=1, the system considers only communication performance. It is necessary to adjust the trade-off factor to balance communication and sensing continuously. The sensing target q∈ℚ={1,…,Q}. C1 is the Quality of Service (QoS) constraint, ensuring the minimum communication rate for each user, C2 ensures that each user belongs to only one cluster, C3 is the total transmission power constraint, $P_{t}$ is the total transmit power of the antennas and C4 is the PAPR constraint, addressing the peak-to-average ratio problem in the sensing waveform. Because of the waveform design problem, F1 is a non-convex problem and is generally difficult to solve directly. This paper decouples the original objective function through quadratic transformations and applies an improved Gaussian clustering algorithm to cluster the users. This reduces the difficulty of SIC decoding and simplifies the objective function. Fractional programming is then used to handle the beamforming design problem at the base station, decoupling the objective function and reducing its computational complexity. Finally, alternating iterative optimization is used to find the optimal variables and obtain a suboptimal solution to the objective function.

## 4. Clustering Algorithm and Beamforming Design

In the communication field, user clustering can manage resources in the network and enhance system fault tolerance. In non-orthogonal multiple access (NOMA), signals are distinguished at the transmitter through power domain multiplexing, enabling the reuse of time–frequency resources. Clustering users with significant channel gain differences can effectively reduce SIC decoding difficulty and intra-cluster interference, thereby improving system performance. Moreover, user clustering facilitates fair resource allocation, ensuring each user receives appropriate communication resources. Therefore, clustering plays a crucial role in overcoming the challenges faced by NOMA, so this section proposes an improved Gaussian Mixture Model (GMM) clustering algorithm for user partitioning.

### 4.1. GMM Gaussian Mixture Model Clustering Algorithm

#### 4.1.1. Basic Concepts of the GMM Algorithm

The Gaussian Mixture Model (GMM) is a probabilistic model that assumes all data points are generated from a mixture of a finite number of Gaussian distributions. Each Gaussian distribution is called a “component”, and each component has its parameters, namely mean and covariance. Additionally, each component has a weight representing the relative proportion of the component in generating the observed data. GMM is a soft clustering method [14], which differs from brutal clustering methods (such as the K-means algorithm [20]). GMM provides the probability of each data point belonging to each component. While maximizing its likelihood function, parameters within each component, such as the mean and covariance matrix, are iteratively updated to find the final clustering results. The following sections will introduce the main concepts of the GMM algorithm:

(1) Gaussian Distribution: The GMM assumes that the data are mixtures of multiple Gaussian distributions. Each Gaussian distribution represents a cluster center of the data.
(2) Mixture Coefficients: Mixture coefficients represent the proportion of each Gaussian distribution in the entire dataset. If there are M Gaussian distributions, there are M mixture coefficients, and their sum equals 1.
(3) Means: each Gaussian distribution has a mean representing the distribution’s and cluster’s centers.
(4) Covariance Matrix: Each Gaussian distribution has a covariance matrix that describes the shape of the data distribution, representing the data variation across different dimensions.

**Figure 2:**
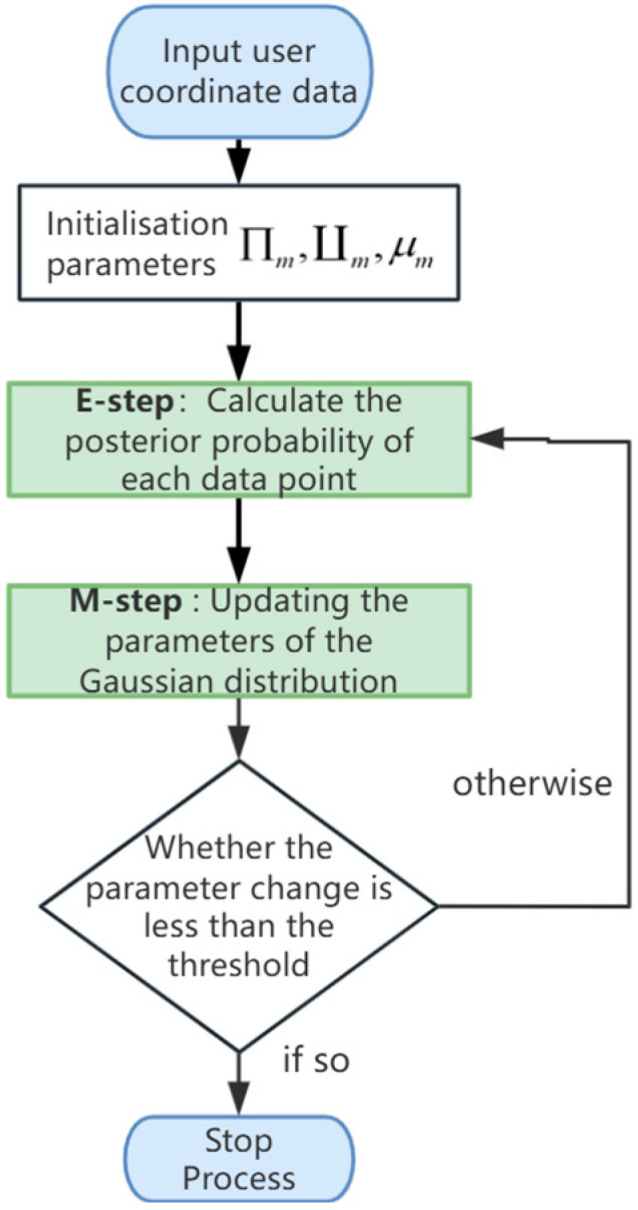
Flowchart of GMM algorithm.

Additionally, the widely used Expectation–Maximization (EM) algorithm is one of the main processes in the GMM algorithm. In the GMM algorithm, the EM algorithm is one of the reasons why Gaussian Mixture Model clustering is so widely applied. This algorithm, which progresses toward maximizing the expectation, allows the GMM to find a local optimum during iterations at least. The general flowchart of the algorithm is shown in Figure 2.

#### 4.1.2. Improved GMM User Clustering Algorithm

Although the GMM algorithm differs from the hard clustering mode of the K-means algorithm, it can cluster data without being limited to spherical or elliptical shapes, resulting in more accurate clustering results. However, it still faces sensitivity issues related to the initial number of clusters, similar to the K-means algorithm. Additionally, in NOMA-ISAC communication, increasing intra-cluster user channel gain differences and balancing communication and sensing performance are critical challenges. Therefore, this paper improves the traditional Gaussian clustering algorithm, making it better suited to the user clustering characteristics of NOMA-ISAC systems.

To address the limitations of algorithm applicability in NOMA-ISAC systems, this paper introduces an improved GMM algorithm. The improved GMM algorithm first reduces sensitivity to the number of centroids by calculating the Sum of Squared Errors (SSE). It then introduces a directional weight centered on the base station, modifying the method for estimating posterior probabilities in the E-Step of the EM algorithm. Additionally, the covariance matrix is introduced to adjust the impact of sensing performance on the overall system. Finally, the likelihood function calculation incorporates a penalty function tailored for clustering in NOMA-ISAC systems. This adjustment influences the final convergence result of the likelihood function, increasing the channel gain differences among users within the same cluster and optimizing the sensing waveform, thereby improving the overall system performance.

#### 4.1.3. Algorithm Flow

Given the user dataset $U=\{u_{1},u_{2},\ldots ,u_{K}\}$ and base station coordinates, with user coordinates and base station location as inputs, the algorithm flow is as follows:

1. m data points are randomly selected as initial clustering centers, i.e., the mean of each Gaussian distribution $\mu_{m}$.
2. The number of clusters M is randomly selected and $SSE=\sum_{m=1}^{M}{\left|u_{i}-\mu_{m}\right|}^{2}$ is calculated. M is the number of clusters and $\mu_{m}$ is the m−th cluster, i.e., the mean of the m−th Gaussian distribution and the point that makes the error squared, and the slope of the SSE mutate is selected as the best positional transverse coordinate as the number of Gaussian distributions M.

**Repeat steps 1 and 2 until the position of the point where the slope changes sharply stabilize.**

Initialize the covariance matrix ${∐}_{m}$ as an identity matrix. To ensure the number of users in each cluster is uniform, initialize the mixture coefficients $\prod_{m}$ to 1/m. Define the vectors from the base station to the cluster centers and user locations as $\vec{b_{\mu_{m}}}$ and $\vec{b_{u}}$, respectively. Calculate the directional weight $\varpi =\frac{\exp\left(\beta \cdot \cos(\theta_{b})\right)}{\exp(\beta )}$, where $\theta_{b}$ is the angle between $\vec{b_{u}}$ and $\vec{b_{\mu_{m}}}$, β is a tuning factor that controls the degree of directional consistency, and $\cos(\theta_{b})=\frac{\vec{b_{\mu_{m}}}\cdot \vec{b_{u}}}{\left\|\vec{b_{\mu_{m}}}\right\|\cdot \left\|\vec{b_{u}}\right\|}$ is obtained through the dot product of the vectors.

**E-Step**

In the E-Step, it is necessary to calculate the posterior probability $l(z_{i}^{m})$ that the user $u_{i}^{m}$ belongs to cluster *m* as follows:

(10) $$l(z_{i}^{m})=\frac{\prod_{m}\cdot \psi (u_{i}|\mu_{m},{∐}_{m})\cdot \varpi_{i}^{m}}{\sum_{j=1}^{M}\prod_{j}\cdot \psi (u_{i}|\mu_{m},{∐}_{m})\cdot \varpi_{i}^{j}}$$

where $\psi (u_{i}|\mu_{m},{∐}_{m})$ is the probability density of data point $u_{i}$ according to the Gaussian distribution of cluster m and is calculated as follows:

(11) $$\psi (u_{i}|\mu_{m},{∐}_{m})=\frac{\exp\left(-1/2{\left(u_{i}-\mu_{m}\right)}^{T}{∐}_{m}^{-1}\left(u_{i}-\mu_{m}\right)\right)}{\sqrt{{\left(2\pi \right)}^{2}\left|{∐}_{m}\right|}}$$

where ${∐}_{m}^{-1}$ is the inverse of the covariance matrix for the m−th cluster and $\left|{∐}_{m}\right|$ is the determinant of the covariance matrix.

**M-Step**

**Update the clustering parameters:** Based on the calculated posterior probabilities $l(z_{i}^{m})$, update the mean $\mu_{m}$, covariance matrix ${∐}_{m}$, and mixture coefficient $\prod_{m}$ for each cluster. To fully utilize the sensing signal’s statistical characteristics and optimize the sensing target’s detection performance, this paper introduces an adaptive covariance matrix update mechanism to achieve the joint optimization of communication and sensing. The updated formula is as follows:

(12) $$\mu_{m}=\frac{1}{\left|C_{m}\right|}\sum_{i=1}^{K}l(z_{i}^{m})u_{i}$$

(13) $${∐}_{m}=\frac{1}{\left|C_{m}\right|}\sum_{i=1}^{K}l\left(z_{i}^{m}\right)(u_{i}-\mu_{m}){\left(u_{i}-\mu_{m}\right)}^{T}+\varepsilon w_{i}w_{i}^{H}$$

(14) $$\prod_{m}=\frac{\left|C_{m}\right|}{K}$$

where ε is the weight parameter used to adjust the impact of the sensing signal on the intra-cluster distribution.

**Check for convergence:**

In this step, it is necessary to calculate the value of the likelihood function and check whether it has converged, i.e., whether its change is below a preset threshold or if the maximum number of iterations has been reached. Additionally, a penalty function is introduced in the algorithm to encourage finding user clusters that fit the clustering characteristics of the NOMA-ISAC system. The penalty function is defined as follows:

(15) $$\xi =\sum_{m=1}^{M}\frac{1}{\left|C_{m}\right|\left(\left|C_{m}\right|-1\right)}\sum_{i,j\in C_{m}}dist(u_{i},u_{j})+\sum_{q\in \mathbb{Q}}\tau (1-p(\theta_{q}))$$

where $dist(u_{i},u_{j})$ is the Euclidean distance between data points $u_{i}$ and $u_{j}$. τ is the perceptual weight parameter. The likelihood function is given by the following equation:

(16) $$L=\log_{2}Ρ(U|\prod ,\mu ,∐)\;=\sum_{i=1}^{K}\log_{2}(\sum_{m=1}^{M}\prod_{m}\psi (U_{i}|\mu_{m},{∐}_{m}))$$

where U is the set of all user coordinates. The improved objective likelihood function is given by the following equation:

(17) $$L^{\prime }=L-\alpha \xi$$

where α is the regularization coefficient. The larger the model, the more significant the increase in the difference in user channel gains within the cluster, which may result in uneven mixture coefficients across clusters.

**Repeat the E-Step and M-Step**

Repeat the E-Step and M-Step until the objective function converges or the predetermined maximum number of iterations is reached. Otherwise, return to the E-Step and continue the iteration process with the updated parameters. The specific method is outlined in the pseudocode shown in Algorithm 1.
**Algorithm 1:** Improved GMM User Clustering Algorithm1: Input user coordinates and base station location, then compute the initial values for the number of clusters M and means μ. $2:\;\mathrm{Initialize}\;\mathrm{parameters}\;\prod_{m}$$\;\mathrm{and}\;{∐}_{m}$. 3:  **Repeat** $4:\;E\text{-}\mathrm{Step}:\;\mathrm{Keep}\;\mathrm{parameters}\;\prod_{m},{∐}_{m},\mu_{m}$$\;\mathrm{unchanged}.\;\mathrm{Compute}\;\mathrm{the}\;\mathrm{posterior}\;\mathrm{probability}\;l(z_{i}^{m})$$\;\mathrm{of}\;\mathrm{user}\;u_{i}$ by (10). $5:\;M\text{-}\mathrm{Step}:\;\mathrm{Keep}\;l(z_{i}^{m})$$\;\mathrm{unchanged}.\;\mathrm{Update}\;\mathrm{parameters}\;\mu_{m},{∐}_{m},\prod_{m}$ by (12), (13), and (14). $6:\;\mathrm{Repeat}\;\mathrm{until}\;\mathrm{the}\;\mathrm{objective}\;\mathrm{likelihood}\;\mathrm{function}\;L^{\prime }$ converges or the maximum number of iterations is reached.

### 4.2. Fractional Programming-Based Beamforming Design

In the optimization problem introduced in Section 2, there is a constraint that each user belongs to a unique cluster. The improved GMM algorithm determines the cluster categories and number of users, addressing the variables $\upsilon_{i}^{m}$ and constraints C2 in the objective function. Therefore, the integrated sensing and communication waveform design problem can be simplified as follows:

(18) $$\begin{array}{l} F1^{\prime }:\underset{w_{i},r_{i}^{m}}{\max}f\left(r_{i}^{m},w_{i}\right)=\rho \sum_{m=1}^{M}\sum_{i=1}^{\left|C_{m}\right|}r_{i}^{m}+(1-\rho )\sum_{q\in \mathbb{Q}}p(\theta_{q})\\ s.t.\;C1:\;r_{i}\geq r_{\min,i},\forall i\in k\\ \;C3:\;W^{H}W=P_{t}\\ \;C4:\;W^{H}E_{n}W\leq \frac{P_{t}\eta }{N},n=1,2,\ldots ,N. \end{array}$$

However, in the waveform design problem addressed in this paper, user and rate optimizations are challenging non-convex problems. Traditional Successive Convex Approximation (SCA) algorithms typically require transforming the problem into subproblems involving additional mathematical techniques and transformation steps, inevitably increasing the complexity of modeling and solving. Therefore, this paper adopts a fractional programming approach to effectively handle the communication rate problem. Reference [15] introduces quadratic transformations to extend single-rate problems to multi-rate problems. Unlike the Charnes–Cooper transformation, this quadratic transformation does not face difficulties with the definition domain of new variables. It can effectively handle continuous problems in beamforming, power control, and other areas in single- and multi-dimensional cases. Additionally, it provides two solution methods, direct methods or solving the Lagrangian dual of the original function in closed form, thereby expanding the approach to solving non-convex problems.

In this paper, the original objective function satisfies the constraints of a concave–convex fractional programming problem [17]. The logarithmic functions in user and rate optimization are non-decreasing. A direct method can be employed to address this multi-rate problem. By introducing auxiliary variables $\lambda_{i}$, the constraint C1 can be reformulated through a quadratic transformation as follows:

C1′ : ri′=BMlog21+2Re{λiHwimhim}         −λiH∑j=1i−1wjmhim2+BMσ2λi≥rmin,i

where $\lambda_{i}^{H}$ is the conjugate transpose of $\lambda_{i}$. Therefore, the optimization problem (18) can be reformulated as follows:

(19) $$\begin{array}{l} P2:\underset{w_{i},\lambda_{i},r_{i}^{\prime }}{\max}f\left(r_{i}^{\prime },w_{i},\lambda_{i}\right)=\rho \sum_{m=1}^{M}\sum_{i=1}^{\left|C_{m}\right|}r_{i}^{\prime }+\left(1-\rho \right)\sum_{q\in \mathbb{Q}}p\left(\theta_{q}\right)\\ s.t.\;C1\prime \;:\;r_{i}^{\prime }\geq r_{\min,i}\\ \;C3,C4\\ \;C5:\;\lambda_{i}\in {\mathbb{C}}^{1} \end{array}$$

By applying a multidimensional quadratic transformation to decouple the numerator and denominator, the optimization problem (18) is converted into the above optimization problem (19). The SINR ratio term is transformed into a concave function concerning $w_{i}$. Given the non-decreasing and convex nature of the outer logarithmic function when the auxiliary variable $\lambda_{i}$ is fixed, the optimization problem (19) becomes a problem convex relating to $w_{i}$. According to the definition of concave-convex fractional programming, an iterative optimization approach is employed. Fixing $w_{i}$ and taking the partial derivative of the reconstructed function pertaining to $\lambda_{i}$, setting the derivative to zero yields the optimal $\lambda_{i}$ as follows:

(20) $$\lambda_{i}^{*}=\frac{w_{i}^{m}h_{i}^{m}}{\sum_{j=1}^{i-1}{\left|w_{j}^{m}h_{i}^{m}\right|}^{2}+\frac{B}{M}\sigma^{2}}$$

When $\lambda_{i}$ is fixed, the entire integrated sensing and communication beamforming design problem becomes a convex optimization problem. The beamforming vector $w_{i}$ can be solved using convex optimization methods, as detailed in Algorithm 2.
**Algorithm 2:** Iterative Optimization Algorithm with Fixed $\lambda_{i}$$1.\;\mathrm{Initialize}\;a\;\mathrm{feasible}\;\mathrm{value}\;\mathrm{for}\;w_{i}$ **2. Repeat** $3.\;\mathrm{Update}\;\mathrm{the}\;\mathrm{value}\;\mathrm{of}\;\lambda_{i}$ using Equation (20). $4.\;\mathrm{With}\;\lambda_{i}$$\;\mathrm{fixed},\;\mathrm{solve}\;\mathrm{the}\;\mathrm{convex}\;\mathrm{problem}\;(19)\;\mathrm{to}\;\mathrm{update}\;\mathrm{the}\;\mathrm{value}\;w_{i}$. **5. Until**
 the function f in problem (19) converges.

## 5. Simulation Experiments

To validate the effectiveness of the proposed algorithms and methods, we conducted a detailed numerical simulation of the clustering performance of the improved GMM algorithm and its impact on the system performance within the NOMA-ISAC framework. The communication area is assumed to be 100 × 100 m^2^, with the base station located at the center. The communication users are randomly distributed around the base station, while the sensing targets are located at the edge of the region at angles of 45° and −45°. The other parameters are shown in Table 1.

**Figure 3:**
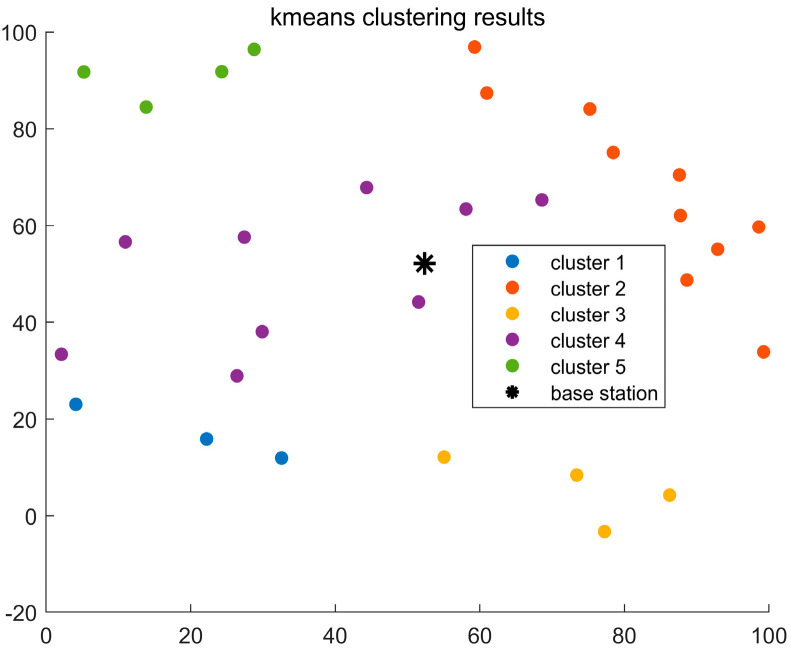
Clustering results of the K-means algorithm.

**Figure 4:**
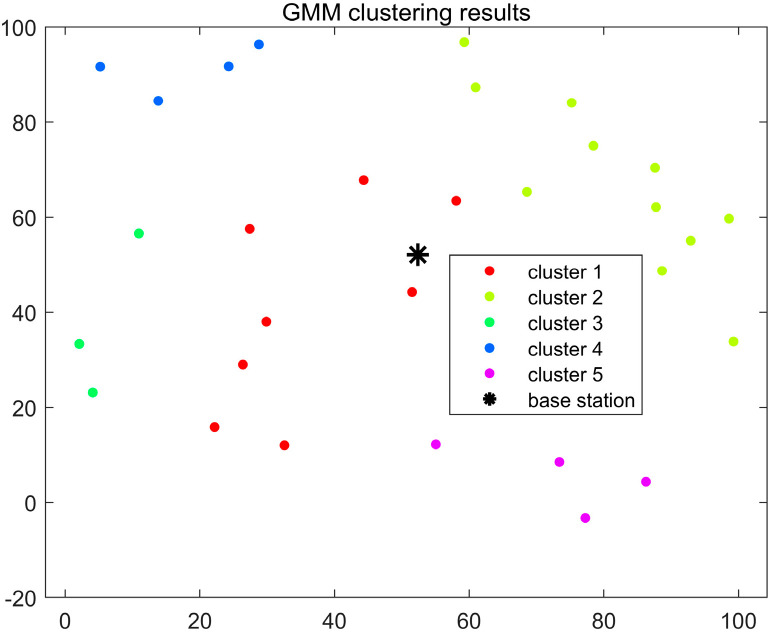
Clustering results of the traditional GMM algorithm.

**Figure 5:**
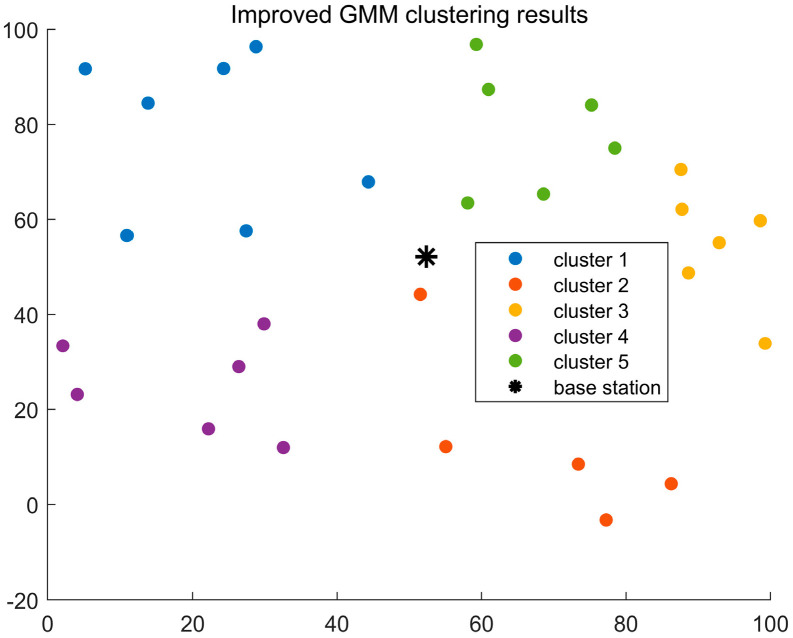
Clustering results of the improved GMM algorithm.

To demonstrate the clustering performance of the improved GMM algorithm, we compare it with the original GMM clustering algorithm and the K-means clustering algorithm. In this comparison, parameter a is set to 0.4 and b to 1.2. Based on the initialization of the improved GMM algorithm in Section 4.1.3, the optimal number of clusters is determined. For the K-means algorithm, the initial K value is set to 5. Due to the sensitivity of the K-means algorithm to the initial positions of the cluster centers, as shown in Figure 3, the user distribution in Cluster 2 is scattered and not concentrated. This is because the initial cluster centers in the K-means algorithm are randomly chosen, resulting in suboptimal clustering. In Figure 4, it is clear that the GMM algorithm can accommodate user clusters of various shapes and sizes. However, in the clustering results of both algorithms, the number of users in each cluster is not evenly distributed, and there are significant weight differences between clusters. Therefore, both algorithms perform poorly for user clustering in NOMA communication. Figure 5 shows the clustering effect of the improved GMM algorithm. It can be seen that the algorithm clusters users into strip-like categories centered on the base station. Each cluster has a weight close to 0.25, and the number of users in each cluster is more evenly distributed than the K-means and traditional GMM algorithms. Considering the base station’s location, this algorithm is more suitable for user classification in NOMA communication. Additionally, Figure 5 shows that Cluster 4 has fewer users than the other clusters. This is because when α>0 is relatively large, the algorithm emphasizes intra-cluster diversity, which may cause the mixture coefficient $\prod_{m}$ of each cluster to become uneven. During iteration $\prod_{m}$, introducing directional weights makes one cluster’s mixture coefficient more significant than the others, resulting in a smaller proportion for another cluster.

**Figure 6:**
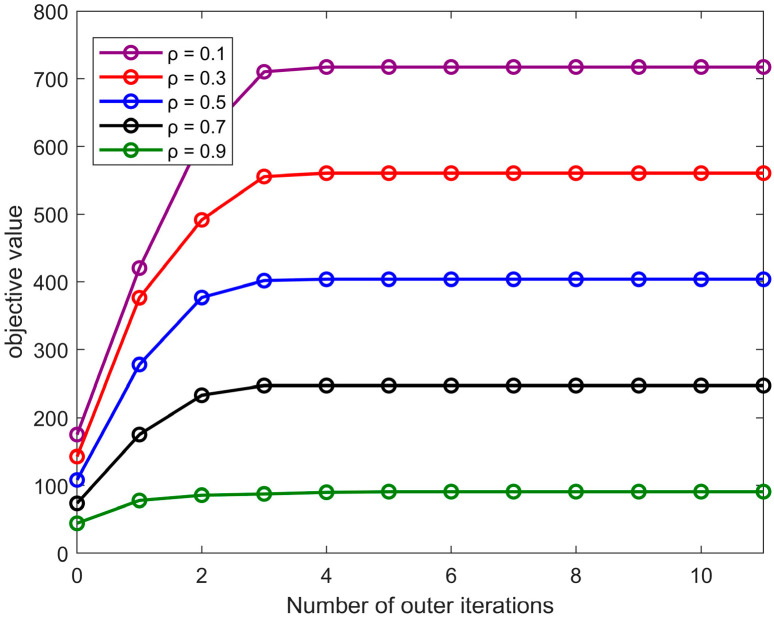
Convergence curves of objective values under different ρ values.

The objective function of this paper maximizes the weighted sum of communication rate and sensing power within the NOMA-ISAC system. The goal is to maximize this function value without compromising user communication quality. Therefore, the choice of the trade-off factor in the objective function is crucial, as it must balance both user communication rate and sensing performance. Figure 6 shows the relationship between the number of iterations and the objective function value under different values. It can be observed that since the sensing power has a higher value compared to the communication rate, the objective function value increases more significantly as ρ decreases. However, regardless of the value ρ, the algorithm consistently converges to a stable function value after about four iterations.

**Figure 7:**
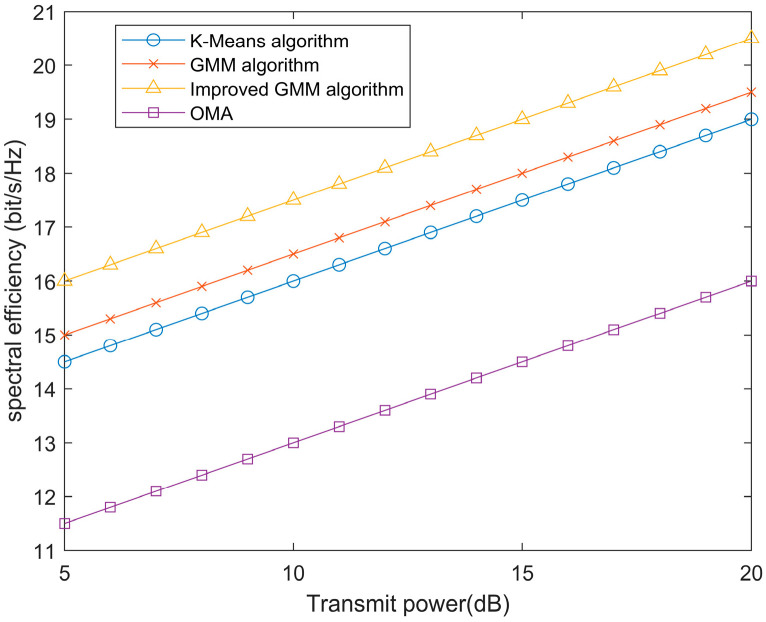
Spectral efficiency vs. transmission power for different clustering algorithms.

To further illustrate the impact of the proposed improved GMM algorithm on the performance of the ISAC system under NOMA communication, we compared the spectral efficiency of the system using the K-means algorithm, traditional GMM algorithm, and OMA-ISAC with the spectral efficiency of the improved GMM algorithm. The improved GMM clustering algorithm introduces a penalty function and direction weights that enhance the diversity of users within clusters and provide an advantage in signal processing at the receiver end during SIC compared to the base station’s strip-like cluster categories. Figure 7 shows that the improved GMM algorithm provides a superior increase in spectral efficiency for users compared to the other two algorithms.

**Figure 8:**
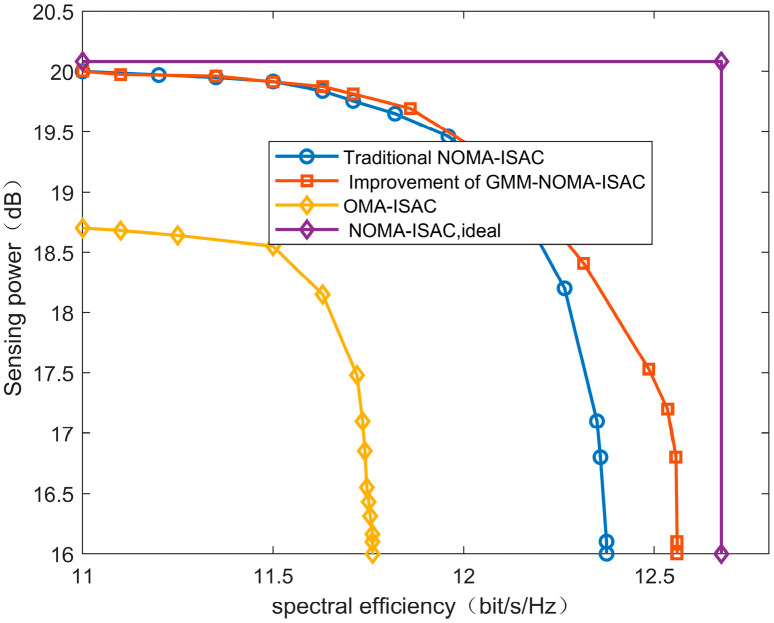
Performance trade-off comparison under different mechanisms.

In NOMA communication, received signals are affected by interference from other users and noise, so the receiver needs to perform SIC (successive interference cancellation) to eliminate interference. The transmitter allocates power based on the channel differences between users, making it easier to decode signals from users with more considerable channel differences. Figure 5 has verified that the improved GMM clustering algorithm is more effective in identifying strip-like clusters with more significant user differences. To demonstrate the performance improvement of this algorithm for the ISAC system, we compare the ISAC system using this algorithm with the NOMA-ISAC system, OMA-ISAC system, and the ideal NOMA-ISAC system. As shown in Figure 8, in the ideal NOMA-ISAC system, communication and sensing operate independently and do not affect each other. As shown in Equation (9), the ideal spectral efficiency and sensing power are obtained by excluding sensing and communication from the problem. Since NOMA allows multiple users to communicate simultaneously on the same frequency band, it can significantly improve spectrum utilization, thereby supporting more user connections and enhancing the spectral efficiency of the ISAC system. In contrast, the OMA-ISAC system requires orthogonal resources, resulting in reduced system capacity and spectral efficiency compared to the NOMA-ISAC system, with a significant gap compared to the ideal NOMA-ISAC system. The introduction of the improved GMM algorithm into the NOMA-ISAC system aims to enhance its channel quality, allowing different users within the same cluster to be effectively distinguished. This leads to a 4.3% increase in spectral efficiency compared to the OMA-ISAC system, further improving the overall system performance.

**Figure 9:**
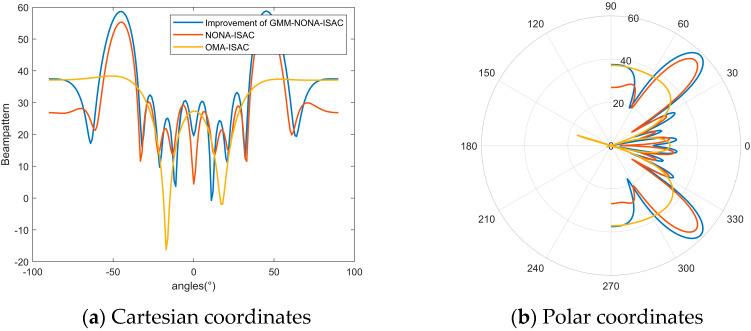
Beam strength curves in different coordinate systems.

Figure 9a and Figure 9b show the beam intensity of the base station’s transmitted beam as a function of angle in Cartesian and polar coordinates, respectively. In the Cartesian coordinate system, the beam energy of the NOMA-ISAC system is relatively concentrated in the target sensing directions of 45° and −45°, indicating that under the QoS constraints of numerous communication users, the system can still maintain good sensing performance. However, due to interference between multiple users, the base station’s beam covers multiple clusters, preventing the absolute concentration of beam intensity in the target sensing direction. The polar coordinates also show the beam intensity in the target sensing direction. It can be observed that in the OMA communication-based ISAC system, there is a significant energy leakage in the target direction. This occurs because as the number of communication users increases, the system becomes overloaded, resulting in insufficient spatial degrees of freedom, increased interference between users, and a substantial reduction in beam intensity in the target direction. The improved GMM clustering algorithm effectively reduces the system burden, increases the channel gain difference among users within clusters, and further enhances the SIC decoding efficiency. Compared to traditional communication–sensing integrated systems, beam intensity improves by approximately 5.4%, and in NOMA communication, users can share spectrum resources through power multiplexing, thus enhancing spectral efficiency.

## 6. Conclusions

To enhance the performance of the ISAC system, this paper employs non-orthogonal multiple access (NOMA) and proposes an improved GMM user clustering algorithm. The goal is to maximize the weighted sum of the communication–sensing objective function. This is achieved by using fractional programming to decouple and convert the original problem into a convex problem for optimization. The simulation results show that the NOMA-assisted ISAC system can reduce user interference during system overload. The improved GMM user clustering algorithm retains the algorithm’s ability to capture the distribution of users within clusters and enhances the system’s ability to optimize the sensing target signal. It yields more uniform strip-shaped clusters, improving bandwidth reuse and reducing serial interference, resulting in a 4.3% and 5.4% increase in spectral efficiency and beam strength, respectively, compared to traditional ISAC systems. Additionally, fractional programming effectively enhances power gain and channel gain, further improving the performance of the ISAC system.

This study only considers the case where the base station beam covers multiple-user clusters. Future research will design base station beams tailored to each user cluster’s characteristics.

## Figures and Tables

**Table 1:** Experimental simulation parameter settings.

Simulation Parameters (Units)	Values
Number of Users K	30
Number of Sensing Targets Q	2
Number of Antennas N	4
System Bandwidth B (MHz)	10
Total Transmit Power $P_{t}$ (dB)	25
$\mathrm{Noise}\;\mathrm{Power}\;\sigma^{2}\left(\mathrm{dBm}\right)$	−174
$\mathrm{Minimum}\;\mathrm{Rate}\;r_{\min}$ (bit/s)	1
Maximum Iterations for Improved GMM	1000
Maximum Iterations for Fractional Programming Algorithm	10

## Data Availability

Date are contained within the article.
